# Supplementary figures and images for: Drosophila larvae form appetitive and aversive associative memory in response to thermal conditioning
Source: PLoS One. 2024 Sep 24;19(9):e0303955. doi: 10.1371/journal.pone.0303955 (PMC11421805; doi:10.1371/journal.pone.0303955)

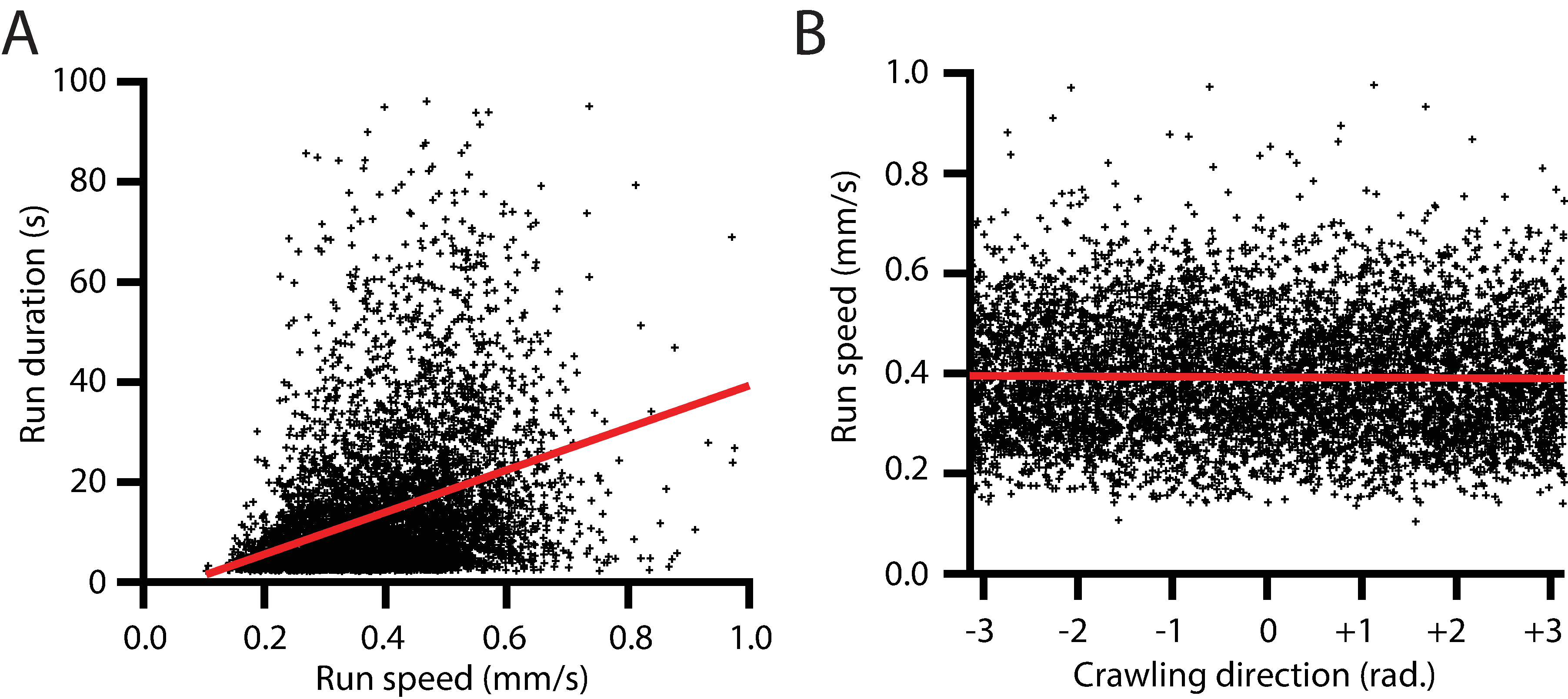

Supplement: S1 Fig — Scatter plots from non-conditioned control larvae crawling on plain agar gel with a linear gradient (same experiments as Fig 3, topmost gray bar). Each individual “run” from all trajectories (N = 240 larvae) is considered separately, and its duration, average speed, and average crawling direction are extracted. Average turn rate is the inverse of the average run duration. A: Run duration vs. run speed, showing a significant correlation. A linear fit to the data provides a slope, which can then be used to regress the effect of speed out of run duration data. B: Run speed vs. crawling direction. These two quantities are not correlated, seen here for neutral thermotaxis crawling on a linear gradient, but also true when thermotaxis is present (see every entry in the “SPEED” column of S3 Fig). (TIF) [file pone.0303955.s001.tif]

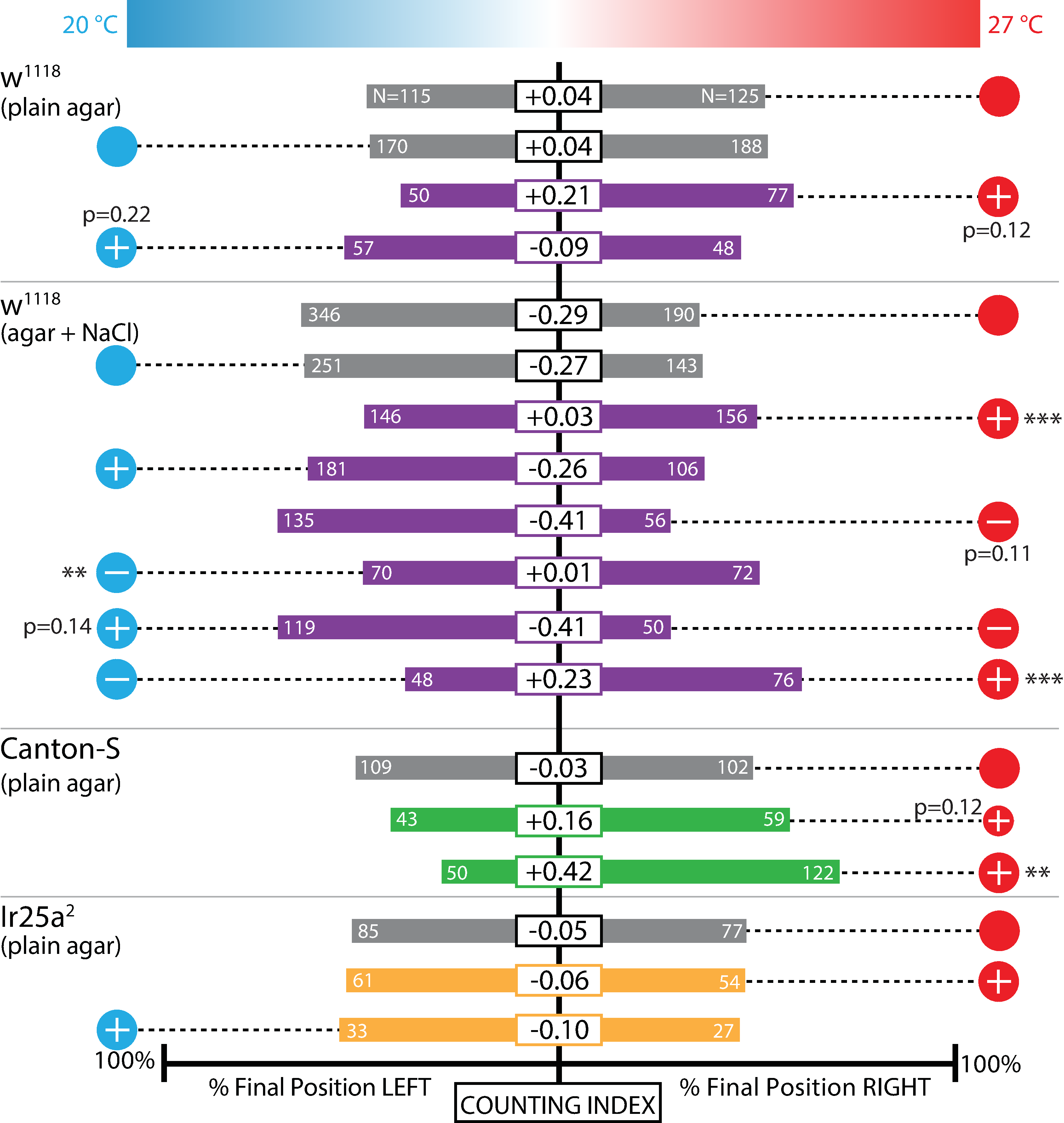

Supplement: S2 Fig — This is an alternate method of determining preference, based solely on the final location of each larva after 10 minutes of activity on the linear gradient testing arena. The experiments are the same as used in Figs 3–6. Horizontal bars indicate the percentage of animals with final position to the LEFT (NL) or RIGHT (NR) of the starting location, and the number in the white rectangle for each experimental condition indicates the preference index, which is (NR − NL)/(NR + NL), or equivalently, the difference between the right and left fractions. Significance tests are with respect to the control (gray bar) group for that strain and type of experiment. * indicates p < 0.05, ** indicates p < 0.01, *** indicates p < 0.001, Fisher’s exact test. (TIF) [file pone.0303955.s002.tif]

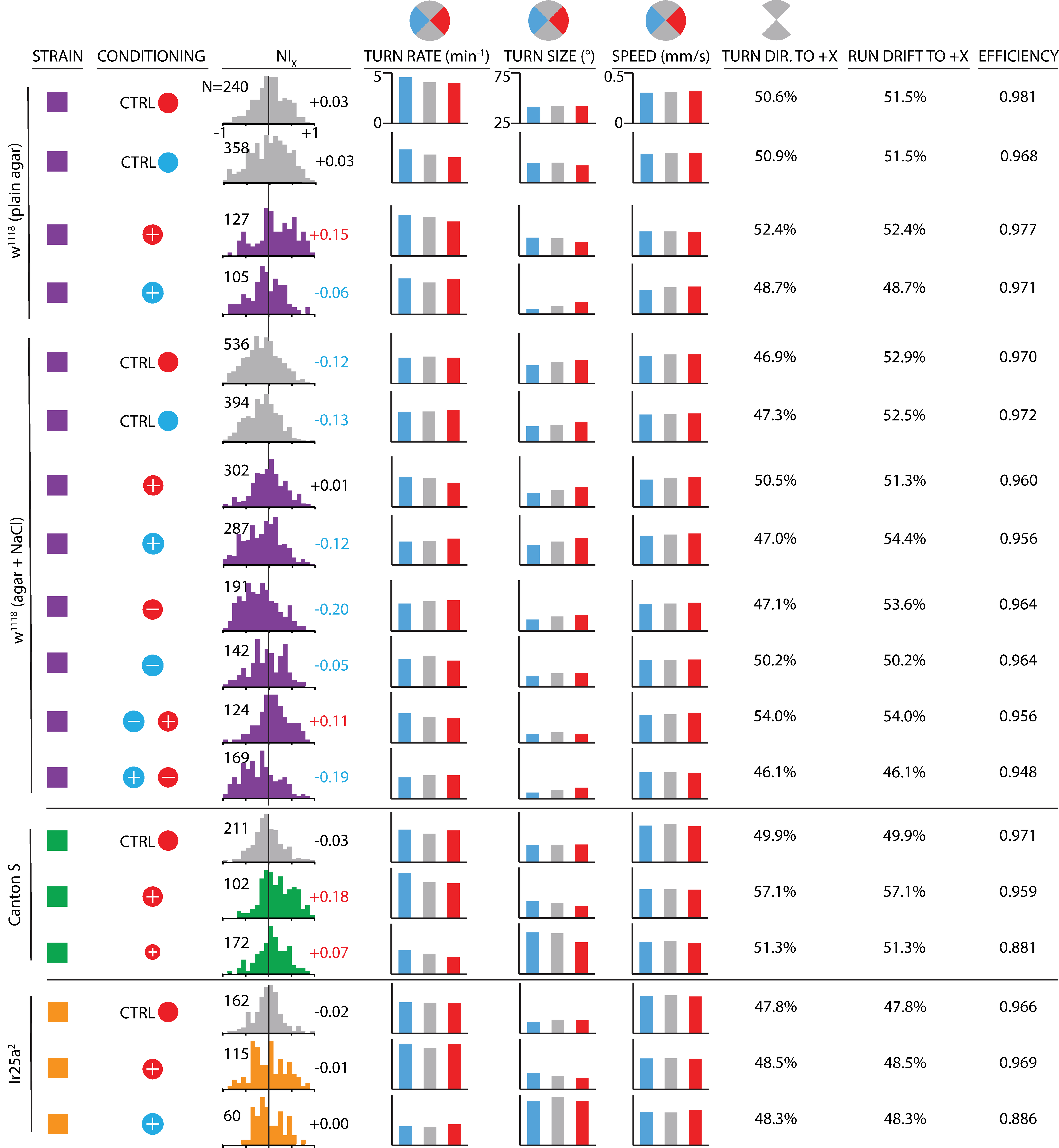

Supplement: S3 Fig — The experiments are the same as used in Figs 3–5, and used the linear thermal gradient for testing. Strain is indicated by a colored square: w1118 (purple), Canton-S (green), and Ir25a2 (orange). The conditioning protocol prior to thermotaxis testing is indicated by colored circles with + or − symbols as described in Fig 2. The navigation index NIx is the average of NIx for each individual larva, with the full distribution shown as a histogram and the average printed adjacent to it (red text indicating NIx > 0.04 and blue text NIx < −0.04). Turn rate, turn size, and speed are shown as a function of crawling direction, sorted into the wedges pictured above the columns (blue for the −x direction, red for + x direction, gray for + /−y direction). Turn direction to + X indicates the percentage of turns made following a run headed in the + /−y directions that point the larva to the warm (+ x) side of the gradient. Similarly, run drift to + X indicates the percentage of runs that drift towards the warm (+ x) side of the gradient. Finally, efficiency indicates how straight are the runs during the experiment set. For each run, efficiency is the ratio of the displacement to the path length, each animal’s efficiency is the average of its run efficiencies, and the number in the table is the average of all the animals’ run efficiencies. (TIF) [file pone.0303955.s003.tif]
