# Supplementary material for: Drosophila larvae form appetitive and aversive associative memory in response to thermal conditioning
Source: PLoS One. 2024 Sep 24;19(9):e0303955. doi: 10.1371/journal.pone.0303955 (PMC11421805; doi:10.1371/journal.pone.0303955)
Supplement: Attachment — Submitted filename: PLOSONE_reviews_resubmission2.pdf [file pone.0303955.s004.pdf]

RESPONSE TO REVIEWERS [PONE-D-24-18023] [Polizos, Dancausse, Rios, Klein]

(Original reviewer comments in italics, our responses in normal text)

REVIEWER #1:

The authors built a Drosophila larval learning paradigm to test the roles of temperature in associative learning, and they successfully demonstrated the associative learning using thermal cues as conditioned stimulus to pair with gustatory unconditioned stimulus. In addition, the author developed analysis pipelines for the locomotion trajectory data, especially for speed and turns, to depict the movement patterns of the larvae in learning and memory.

In general, there are several major questions the authors need to address before publication:

1. For the testing part, did the authors used all three arenas or just one of them for all experiments (appetitive, aversive, and double unconditioned stimulus association)?

The linear gradient was our primary arena for experiments, as it is a simpler geometry and the stimulus experienced by the crawling larvae is more straightforward (e.g., the rate of change of temperature is constant for straight runs). We used the radial gradient arena for controls and three of the four single-conditioning protocols (FRU-27C, NaCl-20C, NaCl-27C), in both thermal geometries (warm inside/cold outside and cold inside/warm outside). This was previously stated in the second paragraph of the Radial Gradient subsection in Results, but we have added a direct statement about the types of experiments we did NOT perform on this setup. We have also adjusted Figure 2 (the protocol schematic figure) to emphasize the linear gradient as the testing arena, since every experiment type was tested there.

If they have the data for all three arenas, is there any difference between them?

For the conditioning protocols that were tested on all three arenas (FRU-27C, NaCl-20C, NaCl-27C):

(1) There was no difference for NaCl-27C

(2) FRU-27C was the same in the linear gradient and the radial gradient with 27C on the perimeter, but we did not observe the effect when 27C was in the center. A possible explanation for this was given already in the original submission (in the Results section), and has now been mentioned again in a greatly expanded section in Discussion as well.

(3) NaCl-20C did not show aversive associative memory on the radial gradient. The aversive behavior is seen in the linear gradient, and is statistically significant, but is a more subtle effect, and we suspect was not observed in the radial gradient arenas due to a lower number of larvae tested and because the more complex geometry may mask more subtle navigation.

All three of these comparisons are now more clearly listed and explained in the radial gradient subsection in Discussion.

Is there anyone worked better, especially for the aversive association?

With new experiments added in this resubmission, establishing aversive association worked more clearly in the linear gradient, which both has a simpler geometry and a large number of new experiments performed in order to be statistically convincing.

If the authors could list this information clear, it would be helpful to understand their conclusions.

Absolutely, and we thank the reviewer for asking about this. The differences between results in the various geometries are now extensively laid out in the Discussion section.

2. For the aversive associative learning part, especially Figure 7, personally I do not think the authors can draw the conclusion that "larvae treated with aversive NaCl Conditioning do form negative associative memories with a thermal conditioned stimulus". The results need to show statistically significant difference between the trained and control groups. Indeed, the final number counts in S2 can be used to support the authors' conclusion, and the radial gradient results really showed associated aversive learning.

[Note that the results in question are now part of Figure 6]. We agree with this assessment and in response have recorded a large number of new experiments, now including two types of control data (at the request of the other reviewer), and more experiments with the aversive NaCl conditioning. We now clearly show that aversive conditioning "works" in both directions, causing larvae to move away from 27C more strongly than controls (for NaCl-27C pairing) and away from 27C <u>less</u> strongly (for NaCl-20C).

However, the authors may need to consider organizing the whole manuscript with a same testing method, the others can be used in supplementary data.

I believe this would no longer be necessary given the changes from the previous item? We note that we have revised the two supplementary figures (S2 and S3) to reflect all the new experiments.

Maybe the low temperature reduces the salt sensation (for the control of aversive association), as the authors explained, so other aversive unconditioned stimulus may be taken into consideration. The authors also can try other training paradigms, such as electric shock or bitter taste solutions for unconditioned aversive stimulus, which are also commonly used.

This is an excellent point, and we actually tried both of these in our preliminary experiments before settling on NaCl (despite the unexpected negative thermotaxis in the control groups). We tried quinine as a bitter taste US, but could not reliably see a change in navigation in conditioned larvae. And we tried electric shock, which also produced inconclusive results, and had the added issue where electric current would heat the larvae, not ideal when temperature was our CS. It does seem important to mention this in the manuscript, so we have added sentences similar to what is written here to the section where NaCl protocols are first introduced in the Methods section.

3. Figure 6, the authors draw a conclusion "these results suggest that anosmic larvae are able to form association between tastant and temperature despite a loss of olfactory sensation". The authors may re-phrase this sentence, as the statistics of this set of experiments is not significant difference.

We agree, that statement was not accurate given the statistical significance we calculated. We ran many more experiments with hundreds more larvae since our initial submission, including additional controls, and the results are still inconclusive, if anything even less consistent. We have decided to remove the old Figure 6 and discussion of Orco2 mutant experiments. The result concerning anosmic larvae was quite secondary to the main conclusions of the manuscript, and we don't have sound explanations for the results. The relationship between taste and smell sensing is likely more complicated than we first thought, and I think beyond the scope of this manuscript.

4. For double unconditioned stimulus part, could this result reflect appetitive association with FRU plus the effect of NaCl during testing? The authors may consider another control group: FRU-Temperature conditioning, while testing on the NaCl agar with temperature gradients, then compare with the double unconditioned stimulus results.

This was a great suggestion, we are glad you provided it! We ran both FRU-27C and FRU-20C conditioning experiments, testing on an agar+salt gel. These can be seen in Figure 6B in the resubmitted manuscript. We used nearly 600 larvae total in these new experiments. The FRU-27C conditioning experiments moved towards the conditioned temperature more strongly than the corresponding control group did (also tested on the same salt gel). The FRU-20C group did not.

The double conditioned larvae appear to navigate more strongly in the corresponding direction than the single FRU conditioned larvae, for both 20C and 27C conditioning, but neither is significant to within $p<0.05$ (the p-values are both <0.1, so they are probably different, just not with 95% confidence).

In addition to the figure update, main body text has been adjusted to include the new experiments.

There are some minor questions below:

1. Line 22 "due their to" should be "due to".

Fixed, thank you.

2. In Figure 1, a legend is needed for the temperature heat map.

Temperature labels have been added to the heat maps for clarity, and colored scale bars have been added to both Temperature vs. position (x in panel A, r in panel B) graphs to make the scale more explicit.

3. What do the traces in the inset of Figure 1 mean, are they representative locomotion traces, or the temperature measurement locations?

They are representative trajectories for the larvae. A sentence has been added to the Figure 1 caption to clarify.

4. Line 231, "temperature-odor pairing" should be "temperature-taste pairing".

This mistake has been removed while rearranging the sentences in this section.

In general, the authors first achieved to use temperature as a conditioned stimulus in Drosophila larval associative learning in their learning paradigm and arena, and they developed analytical tools and pipelines for learning behaviors.

If the authors could provide more convincing results in the aversive learning part, or they can supply a unified/systematical testing and analysis for all learning paradigms (such as the radial gradient testing for appetitive, aversive, and double unconditioned stimulus learning), this work will be really outstanding in the field of Drosophila larval associative learning.

We hope the new experiments we added are sufficient to meet this criterion. In particular, the aversive learning on the linear gradient should be much more convincing than in the original submission (see Figure 6A vs. Figure 6C). Larvae move less strongly towards (or more strongly away from) normally neutral temperatures following NaCl-temperature conditioning protocols.

REVIEWER #2:

Review on „Drosophila larvae demonstrate associative learning….", by Polizos et al, submitted to PLOS ONE 2024

The authors report experiments in larval Drosophila melanogaster to test for associative learning between high/ low temperature as conditioned stimuli (CSs) and tastant reinforcers (or unconditioned stimuli, USs) (sugar or high concentration salt). I agree with the authors that temperature has some unique advantages as CS and that such a paradigm would be a very useful and significant addition to the available 'toolbox'.

The main claim of the authors, reflected in the title, is that ASSOCIATIVE learning is demonstrated. This, I think, is a justified conclusion for the protocol shown in Figure 2C and the data gathered using this protocol in Figure 8. Here the authors show that when a relatively high temperature is paired with sugar and a relatively low temperature is paired with salt the larvae prefer the high temperature, whereas a preference for the low temperature is observed after reciprocal training (low temperature-sugar, high temperature-salt).

While this leaves open the question which of the four kinds of association indeed contributes how much to the eventual difference in temperature preference, this is convincing evidence that at least one association is formed.

This is convincing in particular because the two groups being compared are equated for all other parameters, such as temperature exposure, sugar- as well as salt-exposure, handling etc.

By the same argument, however, the procedures shown in Figure 2B and the data gathered using that protocol are not convincing: no matter which comparison we look at, there is a confound. For example, considering the first versus the second protocol, any difference in temperature preference during the test could be due not to any association being formed, but to exposure to sugar versus exposure to salt per se (i.e. differences in satiation, osmotic stress, etc).

*Likewise, comparing the first to the third protocol, it might be that larvae that were kept at 27degrees for 3x5min for this reason alone have a different preferred temperature during the test as compared to larvae which were kept 3x5min at 20degrees (**please do see my very last point below**).*

This will come up in the answers to several comments below, but our original submission did a poor job explaining what our controls were. The control group for the original submission was exposed to 27C (alternating with room temperature) prior to testing. And for this resubmission we added many experiments with a second control group that was exposed to 20C prior to testing. We believe this should address any concerns about the protocol comparisons, and we will elaborate in the responses below.

Given the present heading, the advice would be to restrict to that paradigm and data (but please do see my last point below). Whether that data, in w1118 mutants, is enough to warrant publication would be for the editor and the authors to decide.

Personally, I think the scientific community deserves to know that the Figure2C/ Figure 8 paradigm works. If it were my study, I would consider adding wild-type data for that protocol, plus maybe the IR25a mutant and Orco mutant. But again, this is not for me to decide or even advise.

Based on the additional experiments added to this submission (and more accurately describing the controls we already had), we believe the reviewer would agree that the old Figure2C/Figure8 (now Figure 2D/Figure6D) results are convincing, but so are the four single-conditioning experiment types, which all show altered navigation compared to the appropriate control (current Figure 3A and Figure 6C).

Title: Do the larvae demonstrate memory, or is it that the experiments demonstrate memory?

A fair point! Given this mistake, and the more convincing results in the resubmission, we have changed the title of the manuscript.

In the introduction in particular, references may better be restricted to the immediately relevant original papers plus a handful of pertinent reviews.

The large number of citations was there to help establish the state of the field and place our work in that context, but we agree there are probably too many. We have now removed the less relevant references, especially those related to mushroom body circuitry.

Please state more clearly what the room temperature during behavioural experiments was (state is only rearing temperature line 92); maybe add that also in Figure 2?!

Thank you. This has been clarified (it's approximately 24 C in our laboratory in Miami) several places, including in Figure 2A as suggested.

As it is unconventional to perform the final temperature preference test in the presence of a US (for the case of salt), the authors should explain why they do so.

Also a very good point. It turns out this is fairly standard in fly larva learning experiments, and we used this method following advice from other researchers who primarily study larva learning (this is our first publication involving learning and memory). We have added an explanation in the Learning Protocols / "Testing" section of Methods, and included several citations where the researchers use this method.

Maybe better to use either % or M when referring to concentrations?!

Thank you. We now only use wt./vol. % when initially referring to the agar and charcoal concentrations in making gels, as that seems to be the most common way to describe that in other larva papers. Everything else has now been converted to M.

It is not entirely clear to me what the procedure for the "Control" was.

Line 229 reads as if these were only handled, but the legend of Figure 2 reads as if they were exposed to the CSs (a "CS-only" control).

If they were exposed to either 3x5min high or 3x5min low temperature, it would be interesting to see the data separated by these treatments.

If there is no difference, you may show this and argue that, likely but not conclusively, the data in Figure 3 and related instances reflect associative memory…!

We apologize for our poorly explained controls, that was a bad mistake in the original submission.

We have good news and more good news. The good news is that the control group was indeed exposed to the warmer temperature prior to testing in the original submission of the manuscript. And the additional good news is that we have performed many more new experiments where the control group was exposed to the cooler temperature prior to testing. In all cases, these two control groups are not distinguishable from each other (even with very large N for both groups). Now throughout the manuscript conditioned larvae are compared against the more appropriate control (e.g., NaCl-27C conditioned larvae tested on a salt+agar gel are compared to a control group that was exposed to 27C prior to testing and then tested on a salt+agar gel also).

Now all four single-conditioning experiment types yield navigation behavior that is statistically significant from the corresponding control group, so we can more decisively conclude that associative memory can be formed for appetitive and aversive US, paired with either a cool or a warm temperature CS. Our sincere thanks for pointing out this issue and inspiring us to run a lot more experiments in order to better establish our findings.

Specific changes related to this item:

* Figure 2 now explicitly has the Naïve Control protocols shown, and it is also more clear which testing gels were used for each experiment type. Text in the Methods section and the beginning of the Results section has also been updated accordingaly. Figure 2B is new, and the single and double conditioning protocols are now seen in 2C and 2D, respectively. We also altered the color scheme of the blue-to-red gradients throughout the paper, so that the medium room temperature is white now – this makes the protocol schematics more clear because all white circles in the steps now represent room temperature.

* Figure 3 now has its original control group (top gray bar) labeled more clearly, and now a new control group that was exposed to 20C prior to testing. There are now two control histograms shown in Fig. 3B, and Fig. 3C has the correct control group labeled.

* Figure 4 (Canton-S, varying FRU concentration during testing) has the same data, but a more clearly labeled control group (a 20C-exposed control group was not necessary for this data set because there was no conditioning done at 20C).

* Figure 6 (formerly Figures 7 and 8, now combined) includes nearly 3x as many tested larvae for the 27C-exposed control, plus a large number of new 20C-exposed controls. It also contains FRU-27C and FRU-20C single conditioning experiments (as in Fig. 3) but tested now on a salt-agar gradient (at the request of the other reviewer). We also more than doubled the number of NaCl-27C single-

conditioned larvae. Put together, the figure now clearly shows statistical significance for navigation difference following aversive US conditioning compared to controls.
